# The treatment preferences of adolescents and their parents, what has the smile RCT shown us? Smile: specialist medical intervention and lightning evaluation

**DOI:** 10.1186/1745-6215-16-S2-P71

**Published:** 2015-11-16

**Authors:** Lucy Beasant, Nicola Mills, Esther Crawley

**Affiliations:** 1 University of Bristol, Bristol, UK

## Background

Recruitment and retention in adult RCTs is affected by patient treatment preference. Identifying preference, providing evidence-based information, and emphasising the equivalence of treatments has facilitated recruitment to adult RCTs. There is no evidence on how to identify preference in paediatric RCTs.

## Methods

Recruitment consultations were recorded and content analysis was used to analyse the strength of preferences prior to randomisation. In-depth interviews were undertaken with participants (13 mothers and 12 adolescents) in a paediatric RCT (the SMILE RCT). Interview transcripts were analysed thematically.

## Results

Content analysis revealed that most parents and adolescents did not express strong treatment preferences in recruitment consultations prior to randomisation. However, interviews suggested preferences were held but not expressed. We identified the following themes:

1. CONFLICTING PARENT-CHILD PREFERENCE: Most mothers had a stronger and more polarised preference. Adolescents expressed a preference more often if interviewed alone.

2. REASONS FOR PREFERENCE: Mothers wanted: extra treatment and “keeping options open”. Some discussed: “nothing to lose”; financial hardship and education. Adolescents preference was based on; feeling tired; anxiety; meeting new people; treatment was already helping.

3. UNDERSTANDING RANDOMISATION. Most demonstrated a good understanding of randomisation although many used terms such as ‘lucky’ and ‘chosen’.

4. ALLOCATION DISAPPOINTMENT. Parental disappointment led to changes in communication during recruitment consultations.

## Conclusions

Adolescents and parent preferences differ, and adolescents are less likely to express preference if their parent is present. Post randomisation interviews suggest that the majority of mothers were not in equipoise, but their child was still randomised to the RCT.

